# Development of DMPS-EMAT for Long-Distance Monitoring of Broken Rail

**DOI:** 10.3390/s23125583

**Published:** 2023-06-14

**Authors:** Wujun Guo, Zhiyang Yu, Hsiang-Chen Chui, Xiaoming Chen

**Affiliations:** 1 School of Optoelectronic Engineering and Instrumentation Science, Dalian University of Technology, Dalian 116000, Chinahcchui@dlut.edu.cn (H.-C.C.); 2 Signal & Communication Research Institute, China Academy of Railway Sciences Corporation Limited, Beijing 100081, China; yuzhiyang@rails.cn

**Keywords:** EMAT, single-mode A0 wave, constructive interference, signal enhancement

## Abstract

The safety of railway transportation is crucial to social and economic development. Therefore, real-time monitoring of the rail is particularly necessary. The current track circuit structure is complex and costly, posing challenges to monitoring broken tracks using alternative methods. As a non-contact detection technology with a lower environmental impact, electromagnetic ultrasonic transducers (EMATs) have become a concern. However, traditional EMATs have problems such as low conversion efficiency and complex modes, which can limit their effectiveness for long-distance monitoring. Therefore, this study introduces a novel dual-magnet phase-stacked EMAT (DMPS-EMAT) design comprising two magnets and a dual-layer winding coil arrangement. The magnets are positioned at a distance equal to the wavelength of the A0 wave from each other, while the center distance between the two sets of coils beneath the transducer is also equal to the wavelength. After analyzing the dispersion curves of the rail waist, it was determined that the optimal frequency for long-distance rail monitoring is 35 kHz. At this frequency, adjusting the relative positions of the two magnets and the coil directly underneath to be one A0 wavelength can effectively excite a constructive interference A0 wave in the rail waist. The simulation and experimental results show that DMPS-EMAT excited a single-mode A0 wave, resulting in a 1.35-times increase in amplitude.

## 1. Introduction

Railway construction has been extensively implemented worldwide, playing a vital role in facilitating both logistics and personnel transportation as an essential component of the transportation system [1,2]. Rail breakage caused by cracks in the rail waist is one of the most important causes of derailment accidents. Early detection of railway defects is helpful not only in preventing accidents but also in extending the lifespan of the rails. Failure to detect defects in a timely manner can result in unpredictable losses. Therefore, online monitoring of railway defects is a necessary and effective measure [3]. Using track circuits for broken rail monitoring is still widely used among numerous monitoring methods. However, it is susceptible to many factors, such as insulation damage, humidity, etc., which can lead to incorrect alarms, and employing alternative techniques for online monitoring of fractured rails can be challenging [4]. Ultrasonic guided waves exhibit low detection frequencies and can propagate over long distances, making them valuable for monitoring rail systems over extended distances [5].

Laser ultrasound, piezoelectric ultrasound, and electromagnetic ultrasound are the methods used to generate ultrasonic guided waves. Out of these methods, laser ultrasound-guided wave detection is relatively more expensive [6], while piezoelectric ultrasound excitation offers a high ultrasonic signal-to-noise ratio, enabling quick and precise detection of defect size and location. RAILSONIC achieved broken rail detection of CWR using piezoelectric excitation ultrasonic guided wave technology in 2003, with a detection interval as long as 1.75 km [7]. In 2020, Wei et al. [8] developed a long-distance rail detection system using piezoelectric ultrasonic transducers and successfully received a 2.81 V peak-to-peak voltage signal on the rail at a distance of 500 m from the transmitting end to the receiving end. Nevertheless, piezoelectric transducers are susceptible to incorrect detection and unsuitable for long-term rail detection due to their reliance on a coupling agent and susceptibility to environmental factors [9,10]. In the context of intelligent detection for railway tracks, Jun Lu et al. [11] proposed an efficient method called “sceu-net” for detecting damages on high-speed railway tracks. By leveraging machine vision technology, the authors were able to improve the efficiency of track damage detection. On the other hand, Wen Wu et al. [12] presented a defect detection and recognition method for aluminum joints based on guided wave monitoring. Through the utilization of a Bayesian framework and selected damage features, the authors successfully enhanced the computational speed of defect detection. However, the complexity of image analysis algorithms and the large amount of data to be processed can hinder real-time performance, leading to delays in detecting track damages. EMAT is a burgeoning non-contact detection technology that is relatively immune to environmental influences and exhibits potential for long-range rail inspection.

However, the multimodal nature of EMAT presents challenges that significantly impact long-range detection. These challenges include signal superposition, non-uniform energy distribution across different modes, and a decreased signal-to-noise ratio [13]. To tackle these issues, researchers have extensively studied and improved EMATs. Peter Cawley employed numerical simulation methods to investigate the propagation characteristics of Lamb waves and successfully generated single-mode Lamb waves in an aluminum plate [14]. Li et al. employed low-frequency EMAT to generate and detect single-mode Lamb waves in steel plates and pipes of varying thickness, resulting in effective defect detection [15]. Our laboratory achieved single-mode Lamb wave excitation by enhancing the displacement amplitude of the S0 mode and suppressing the displacement amplitude of the A0 mode through an increase in the horizontal Lorentz force, as demonstrated by finite element simulations [16]. Zhang et al. arranged small square magnets with opposite magnetism periodically to significantly enhance the magnetic flux density in the horizontal direction. Through this structure, the amplitude of the A0 wave signal obtained was approximately 9.33 times higher than that of the traditional EMAT [17]. In 2022, Yang et al. proposed a periodic magnetic structure EMAT that significantly enhances the amplitude of the S0 mode Lamb waves by utilizing magnetically periodic arrangements of magnets [18]. Martinho et al. proposed a new design of SH EMAT, which utilizes laterally shifted periodic array magnets to generate unidirectional SH waves. By increasing the number of magnet rows or decreasing the inter-row spacing, this design can reduce the amplitude of backward sidelobes and thus improve the directivity and energy of forward waves [19]. In 2023, Kubrusly studied a method for simultaneously generating two different SH wave modes using a dual-array transducer. They utilized superimposed pure wave modes and unidirectional excitation signals and performed localization in the frequency-wavenumber domain to achieve control over the propagation direction of each wave mode [20]. Alan employed dual periodic permanent magnets (PPM) EMAT to generate shear waves in a single direction and SH guided waves with a single mode by controlling the delay of the excited guided waves [21].

Moreover, the low conversion efficiency of EMAT often results in signals with poor signal-to-noise ratios. Thus, optimizing the design of the transducer is imperative to improve guided signals. Cui used two square magnets of opposite polarities placed side by side, increasing the local magnetic flux density of the cutting coil, which was 1.9 times higher than that of conventional EMAT [22]. In 2022, Qi et al. placed the nickel sheet between the permanent magnet and the coil, achieving an increase in magnetic flux [23]. Zhang et al. developed an enhanced butterfly coil EMAT known as the Three-Square Permanent Magnets of Opposite Polarities (TSPM-OP). This innovation achieved a remarkable 4.97-fold enhancement compared to the conventional butterfly coil EMAT. [24]. Jiang et al. proposed a new non-uniform coil structure for a Raleigh wave EMAT (RW-EMAT), which enables signal compression in the spatial domain. The experimental results showed an increase in received signal amplitude by 2.3–2.6 times [25]. Wang et al. found that the position of the backplate affects the pulse width and amplitude of the thickness measurement signal. Reducing the gap between the coil and the backplate can reduce the pulse width by over 80%, while increasing the gap can increase the signal amplitude by over 300% [26].

The methods for electromagnetic ultrasonic detection of rail tracks can be divided into two categories. The first involves the use of a moving inspection vehicle to achieve electromagnetic rail ultrasonic guided wave detection [27], while the second utilizes SH wave monitoring. However, the latter method has a maximum detection distance of only 1500 mm, which makes it difficult to monitor rail tracks at long distances [28]. Existing literature has shown that there are many studies on the excitation of SH single-mode guided waves, but research on using this method for Lamb waves is limited. When combined with track coils and PPM technology, the amplitude of SH waves is significantly increased. Compared to SH waves with the same parameter matching and detection position, Lamb waves have a larger amplitude, slower attenuation, and longer propagation distance, making them more suitable for long-distance detection. However, as rail tracks are irregular structures, many electromagnetic ultrasonic guided wave methods are difficult to apply because they mostly focus on excitation and near-field detection of regular objects. To address this issue, we developed a DMPS-EMAT based on PPM technology that can excite single-mode A0 guided waves and improve their amplitude. This new technology provides the possibility of realizing remote online detection of rail tracks.

The rest of the paper is organized as follows. The second section introduces the principle of DMPS-EMAT and the principle of A0 wave enhancement using wave phase interference. In the third section, the finite element method is used to simulate the generation and propagation of electromagnetic ultrasonic guided waves in the rail, and the optimization and performance analysis of the transducer are carried out. Finally, experimental verification and analysis are carried out in the fourth chapter.

## 2. Principle and Design of DMPS-EMAT

**Figure 1:**
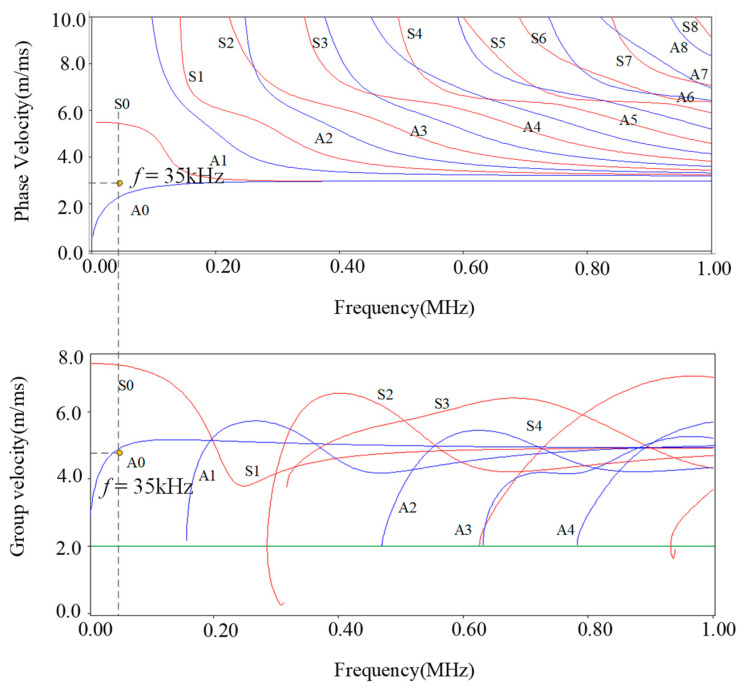
Dispersion curve of the ultrasonic guided wave in the rail waist.

To determine the most effective single-mode Lamb waves for long-distance rail inspection, we used Disperse software (version 2.0) to solve the dispersion curve of a UIC60 rail. The modal analysis of Lamb waves propagation is presented in Figure 1, revealing that the velocity of the A0 mode remains relatively stable in the low-frequency band. Notably, at 35 kHz, the group velocity and phase velocity of the S0 and A0 modes show significant differences, making them easy to separate and identify.

**Figure 2:**
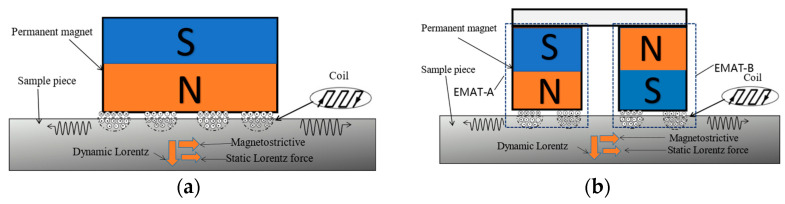
Schematic diagram of (**a**) traditional EMAT and (**b**) DMPS-EMAT.

According to the research conducted by Rose et al. [29], within the frequency range of (0–20 kHz), it is ineffective to acquire long-distance signals due to a low signal-to-noise ratio. On the other hand, at frequencies higher than 40 kHz, multiple waveguide modes are generated during propagation, which makes the detection of signals more difficult due to the increasing attenuation of the waveguide signals. Therefore, we have chosen the frequency range of 20–40 kHz as the optimal interval for signal detection. Within this range, the waveguide exhibits a slow and steady attenuation curve. As the frequency decreases, the transducer size increases. Therefore, we have chosen to utilize the A0 guided wave at 35 kHz for non-destructive testing of rails. For instance, at 25 kHz, the transducer coil spacing is 60 mm, while at 35 kHz, it is 48 mm. Consequently, selecting a frequency of 35 kHz allows for minimal transmission losses, effective transmission over longer distances, and meets the required size criteria. Figure 2a depicts the components of a traditional EMAT, consisting of an excitation coil and a permanent magnet. Typically, the electro-acoustic conversion of EMAT involves three energy transfer mechanisms: Lorentz force, magnetostriction force, and magnetization force. These mechanisms collectively contribute to the generation and propagation of ultrasonic waves in the tested material. Understanding these mechanisms is crucial to optimizing the design and performance of EMATs for various non-destructive testing applications [30,31].

When testing a rail, the Lorentz force, magnetizing force, and magnetostrictive force act in concert, and the process can be expressed by the following formula [32]:

The magnetostriction force $f_{Ms}$ can be expressed as follows (Ms stands for magnetostriction):

(1) $$f_{Ms}=-\nabla_{t}(e^{T}H_{d})$$

where $\nabla_{t}$ is a vector operator that represents the gradient operation with respect to time, $e^{Τ}$ is the inverse piezoelectric magnetic matrix, and $H_{d}$ denotes the dynamic magnetic field strength.

The magnitude of the Lorentz force in a steel rail can be expressed as follows:

(2) $$f_{L}=B_{0}\times J_{e},$$

where $B_{0}$ is the static magnetic field provided by the bias magnetic field, and $J_{e}$ is the induced eddy current density.

The total force can be expressed as follows (where the magnetizing force is small and negligible):

(3) $$f=f_{L}+f_{Ms}$$

The equation for the dynamic magnetic field generated in the coil is as follows:

(4) $$\frac{1}{\mu }\nabla^{2}A-\sigma \frac{\partial A}{\partial t}+\frac{1}{S}\underset{S}{\int \int }\sigma \frac{\partial A}{\partial t}ds=-\frac{i(t)}{S},$$

where *S* represents the cross-sectional area of the coil, *A* is the magnetic vector and conductivity, σ is the stress tensor matrix, and i(t) is the transient excitation current.

When the guided wave signal changes from an electromagnetic signal to the mechanical vibration of rail surface particles, its expression for elastodynamic motion is

(5) $$(\lambda +\mu_{L})\nabla (\nabla •u)+\mu_{L}\nabla^{2}u-\gamma \frac{\partial u}{\partial t}+f=\rho \frac{\partial^{2}u}{\partial t^{2}}$$

where u is the time resonance displacement vector; λ is the Lame constant; $\mu_{L}$ is the Lame constant; ρ is the material density.

The above expression indicates a close relationship between the vibration amplitude of particles and the eddy current density, the magnetic induction intensity of the alternating magnetic field, and the strength of the static magnetic field. As the magnetic field intensity increases or displacement is synthesized through the phase-locked interference of waves, the vibration amplitude of particles also increases. Therefore, for the detection efficiency of EMAT, increasing the magnetic field strength or adopting wave phase-locked interference to synthesize displacement can significantly improve their efficiency.

The design and principle of the DMPS-EMAT are based on the Huygens principle, as illustrated in Figure 2b. The design consists of two permanent magnets and a multi-turn meander coil. The coil is affixed beneath the magnets, with its conductor section positioned directly beneath the two magnets and the spacer section situated under the space between them.

**Figure 3:**
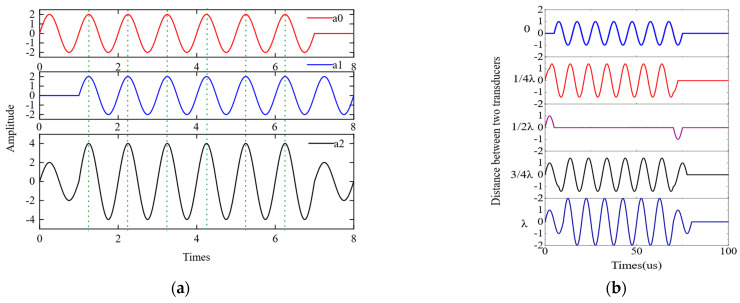
(**a**) Principle diagram of wave synthesis for achieving A0 wave phase interference through magnet-coil distance adjustment; (**b**) waveform synthesis results of magnet-coil distance adjustment at different wavelengths using MATLAB.

By adjusting the distance between the two magnets and the effective conductor of the coil located beneath them, it is possible to guide and concentrate the magnets to generate a magnetic field with secondary constructive interference of the A0 wave. When the distance offset between the Lamb waves excited by coils directly below magnets A and B is one wavelength, the ultrasonic guided waves a0 and a1 generated by the excitation of magnets A and B exhibit a one-cycle phase difference. This method can maximize the constructive interference of the A0 wave, enabling effective excitation and reception of the guided wave, as illustrated in Figure 3a. This results in a combined displacement a2 that is twice the ultrasonic displacement produced by magnets A or B alone. After synthesizing two waveforms with different distances using MATLAB, the resulting waveform diagram is shown in Figure 3b. From the figure, it can be observed that when the distance between the two waves equals one wavelength, the amplitude of the synthesized signal reaches its maximum value, resulting in a more concentrated signal.

## 3. Finite Element Simulation of DMPS-EMAT

### 3.1. Establishment of the DMPS-EMAT Simulation Model

**Figure 4:**
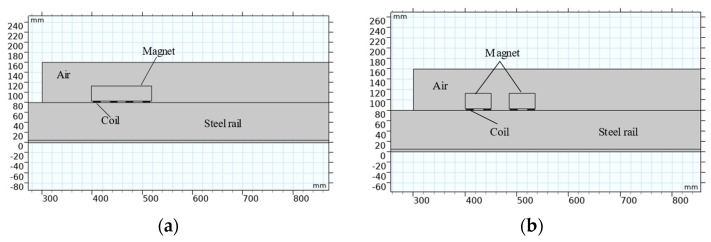
Finite element model of ultrasonic guided wave excitation and propagation: (**a**) traditional EMAT and (**b**) DMPS-EMAT.

The numerical models of the traditional EMAT and DMPS-EMAT were established using the COMSOL Multiphysics software package, and the models are shown in Figure 4 [33,34]. A static bias magnetic field is generated by a permanent magnet, while the coil is positioned at a distance of 0.2 mm from the upper surface of the steel plate. The coil consists of 20 turns of 0.1 mm wide copper wire. In this study, we employed a two-dimensional finite element method to simulate the propagation of electromagnetic ultrasonic guided waves in railway tracks. To facilitate effective wave coupling with the tracks, we utilized free boundaries in the regions where the tracks are in contact with air. In other areas, low-reflective boundaries were employed to mitigate the interference of reflected waves on the distribution of the acoustic field. The coupling between the coil and the system primarily relied on the magnetic field, with the conductivity model used for the conduction analysis and the relative magnetic permeability of the effective medium chosen for the magnetization model. As part of the multi-physics setting, we introduced acoustic structure boundaries and Lorentz coupling. To achieve more accurate solutions, a freely triangular mesh was employed in the track regions, ensuring a minimum of 10 grid elements within each wavelength range. These settings enable us to better simulate the system’s behavior and obtain accurate and reliable results.

Table 1 provides the geometric parameters of the DMPS-EMAT, along with the physical properties of the coils, permanent magnets, and rails used in the finite element model.

The excitation frequency in the model is 35 kHz, and the excitation current injected into the coil is a sinusoidal pulse signal, given by

(6) $$i(s)=\left\{\begin{array}{c} I\times \sin(2\times \pi \times f_{0}\times t)\;\;\;t<=nT0\\ 0\;\;\;\;\;\;\;t>nT0 \end{array},\right.$$

where *n* is the number of pulses of the excitation signal and is defined as 7, *f_0_* is the excitation frequency of 35 kHz, and *I* is the amplitude of the excitation current.

**Figure 5:**
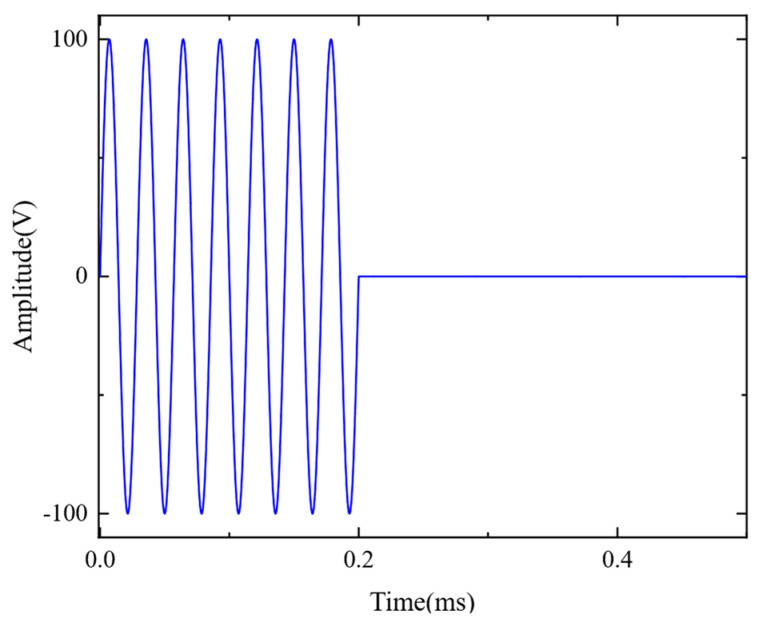
Waveform of the excitation signal.

The ultrasonic waveform of the excitation signal is shown in Figure 5.

### 3.2. Simulation Results and Analysis

**Figure 6:**
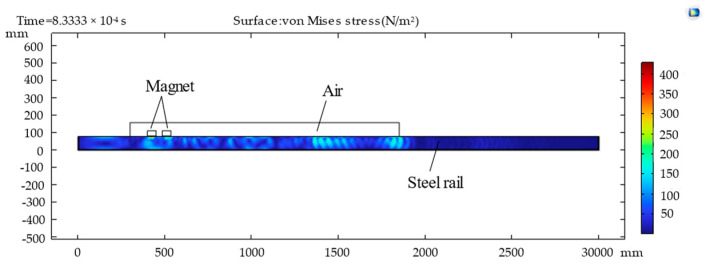
Stress propagation of an electromagnetic ultrasonic guided wave in a rail.

In this work, multi-physics software was used for simulation analysis, in which the structural mechanics module was utilized for the propagation process of the Lamb waves. The calculation process of the multi-physics finite element method model was divided into three parts: simulation of the static magnetic field and the high-frequency AC magnetic field; conversion process of electromagnetic mechanics; and generation and detection process of the Lamb waves. In the transduction region, the electromagnetic mechanics coupled with the Lorentz force are opposite to the detection process and the generation process. The induced electric field was obtained through the cross multiplication of ultrasonic velocity and magnetic flux density. In the process of solving the model, the static bias magnetic field was considered the steady state, and the transient method was used to solve other magnetic fields. Figure 6 illustrates the propagation of the Lamb waves in rails at different times.

**Figure 7:**
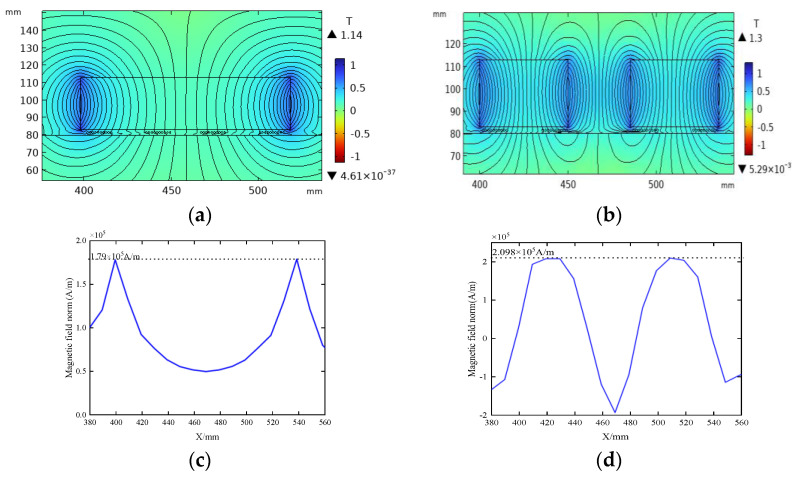
Comparison of flux density distribution: (**a**) magnetic flux density distribution of traditional design, (**b**) magnetic flux density distribution of DMPS-EMAT, (**c**) conventional permanent magnet flux density distribution curve, and (**d**) magnetic flux density distribution curve of DMPS-EMAT.

Figure 7a,b depict the magnetic flux density distribution for two distinct magnetic field configurations, where the residual magnetic flux density is fixed at 1.27 T, and the total magnet size is constant in both cases. Comparative analysis reveals that, in contrast to traditional permanent magnets, the magnetic flux density distribution of DMPS-EMAT is more concentrated and possesses a greater magnitude, as illustrated in Figure 7c. The magnetic flux density distribution of conventional permanent magnets exhibits two peaks at the magnet’s edges, with the minimum value appearing at the center of the magnet. In contrast, in Figure 7d, the magnetic flux density curve of the DMPS-EMAT exhibits a flat peak below each small magnet. Thus, the overall magnetic flux density of DMPS-EMAT surpasses that of conventional permanent magnets. This design approach significantly augments the magnetic flux density on the sample’s surface, thereby reducing the incidence of magnetic flux leakage.

**Figure 8:**
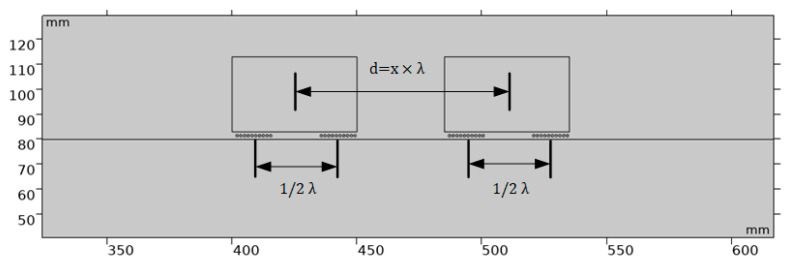
Illustration of exciting single-mode A0 waves by varying the distance (d) between two magnets and the coil located directly below in Comsol, where d is x times the wavelength, and the range of x is 0 ≤ x ≤ 2λ.

**Figure 9:**
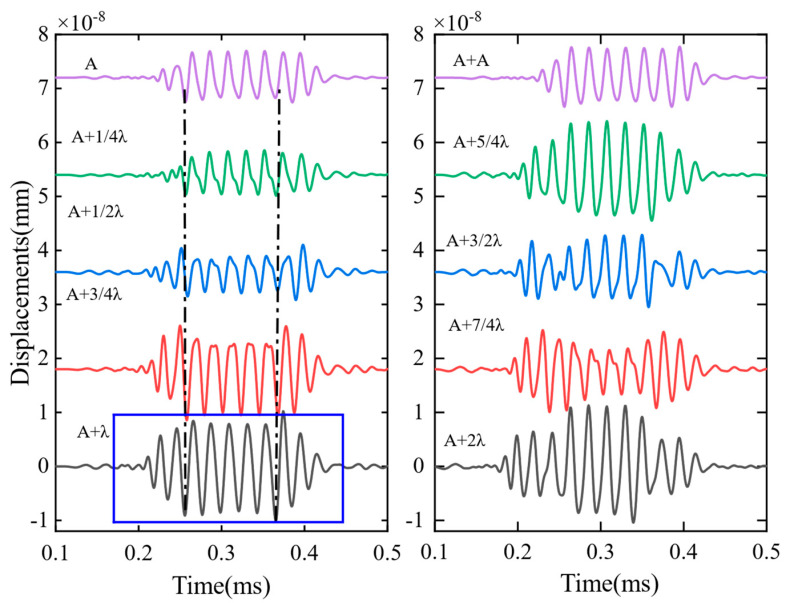
Waveforms of A0 generated by magnet distances at different wavelengths. The symbol “A” represents a meander coil, while “A + A” denotes the arrangement of two coils stacked together. On the other hand, “A + 1/4 × λ” signifies the separation of two coils by a distance of 1/4× times the wavelength.

In order to determine the magnet position suitable for guided-wave constructive interference to excite the A0 wave, it can be optimized by continuously adjusting the distance (d) between the two magnets. As shown in Figure 8, we set the coil spacing directly under each magnet to half the wavelength of the transmitted signal in EMAT (λ/2), simulated the waveform of the A0 wave under different conditions by changing the value of d, and performed comparative analysis to find the A0 wave of constructive interference. The results are presented in Figure 9. We emphasized the signal at a distance (d) equivalent to one wavelength by employing a blue rectangular indicator. At this specific separation between the two magnets, the guided wave signal attains its maximum amplitude, leading to a heightened concentration of energy. This optimization approach enables precise determination of the magnet position, thereby facilitating effective control over the excitation of A0 waves with constructive interference.

**Figure 10:**
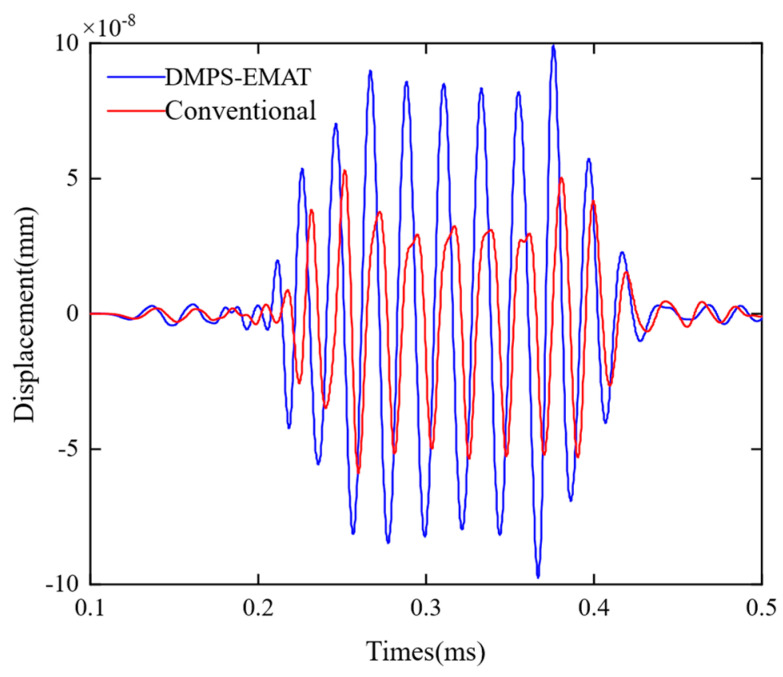
Comparison of received signals before and after transducer optimization in simulation.

To compare the performance of the traditional EMAT and the DMPS-EMAT in exciting A0 Lamb waves, a simulation model was developed to calculate the propagation and displacement detection of the guided wave under identical conditions. The magnets used in the simulation model are 1.2 T rubidium magnets, and the same flexible coils are driven by a 100 V pulse voltage signal. The displacement of the Lamb wave was measured at the same position using a probe located one meter away from the transmitter, as illustrated in Figure 10. The research findings indicate that, under the same experimental conditions, the strength of the Lamb wave signal received by the DMPS-EMAT transducer is 1.63 times greater than that received by the traditional transducer. This result indicates that the DMPS-EMAT transducer has significant advantages in generating and detecting guided waves with higher conversion efficiency.

## 4. Experimental Verification and Result Analysis

### 4.1. Design of Rail Testing Experiment

**Figure 11:**
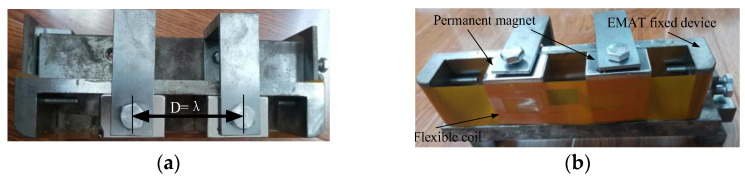
Transducer design. (**a**) Transducer top view and (**b**) transducer front view.

To verify the characteristics of the improved transducer, a non-destructive testing experiment was conducted on a standard UIC60 using the DMPS-EMAT transducer. The transducer was fixed onto the rail waist with a fixture, and ultrasonic guided waves were generated, as depicted in Figure 11.

Usually, when designing a coil, according to the coherence principle of the wave, the line spacing d of the coil is selected to be half of the wavelength λ of the ultrasonic guided wave, and the spatial size of the meander coil is

(7) D=(2N+1)d  n=0,1,2⋅⋅⋅

**Figure 12:**
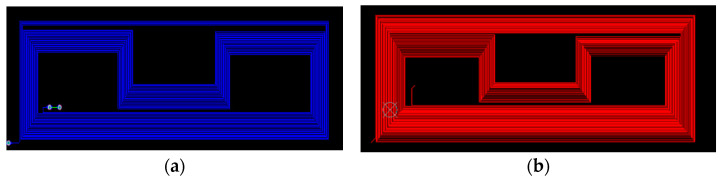
Design of transmitting and receiving coils: (**a**) transmitting coil and (**b**) receiving coil.

According to the measured rail waist thickness, select the phase velocity corresponding to the A0 mode under the appropriate frequency-thickness product. When the rail waist thickness is 20 mm, select 35 kHz as the excitation frequency to find the corresponding phase velocity under the frequency-thickness product, and then the wavelength of the guided wave under this frequency-thickness product is

(8) $$\lambda =\frac{c_{p}}{f},$$

where *c_p_* is the phase velocity of the A0 guided wave. Based on the values of excitation frequency and phase velocity, the corresponding wavelength of the A0 mode at 35 kHz is calculated to be about 81.4 mm with a coil spacing of 40.7 mm (which is λ/2). The double-layer multi-cluster meander coil used in the experiment is shown in Figure 12.

**Figure 13:**
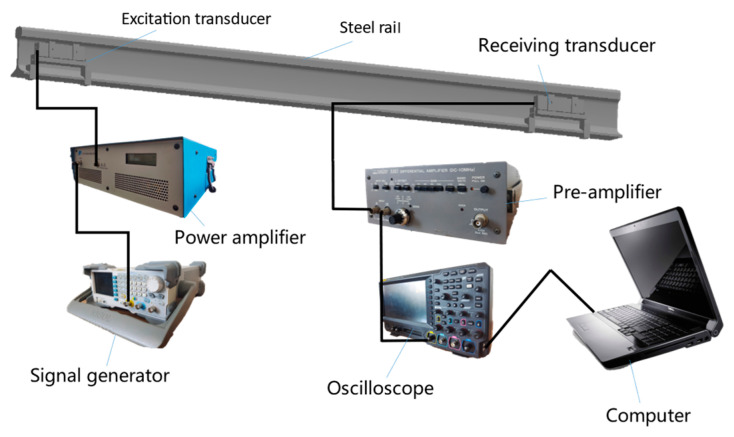
Diagram of the electromagnetic ultrasonic testing system.

Figure 13 presents a schematic diagram of the experimental system utilized for detecting cracks in the rail head. This system employs DMPS-EMAT technology to excite electromagnetic ultrasonic guided waves specifically in the rail head region, enabling rail break detection. Initially, a sinusoidal signal is generated by a signal generator operating at a frequency of 35 kHz and with a peak-to-peak amplitude of 500 mV. The generated signal takes the form of pulse trains consisting of seven pulses with a time interval of 100 ms. Subsequently, this signal is amplified by a power amplifier with a gain of 200 and then fed into the coil of the DMPS-EMAT.

At the receiving end, the DMPS-EMAT utilizes the inverse effect of electromagnetic ultrasonics to capture the vibrational signal of the guided waves on the coil. To optimize signal reception, an impedance matching network is implemented, wherein a 200 μF capacitor is connected in parallel to the ends of the coil to match the impedance between the EMAT and the power amplifier. The signal is further amplified by a pre-amplifier. Moreover, the transmitting and receiving coils are positioned in a straight line parallel to the length of the rail, enhancing the reception of the propagated guided wave energy through the utilization of fixtures.

For the purpose of rail breakage detection, we perform a Fourier transform on the received signal to obtain its maximal spectral value, which is then compared with a threshold set for the scenario without any rail breakage. If the received signal strength is lower than the predefined threshold, the presence of a rail breakage can be confirmed.

### 4.2. Experimental Results and Analysis

**Figure 14:**
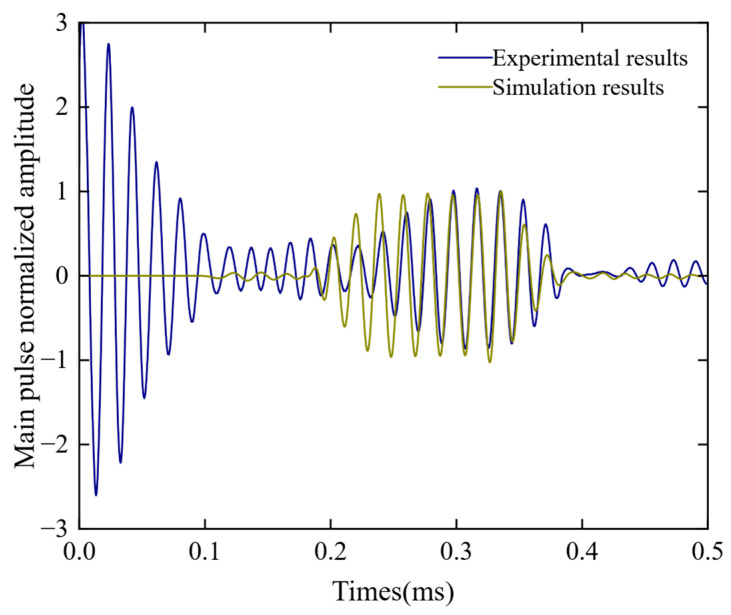
Comparison of simulated and experimental waveforms.

The results of the experiment and simulation were normalized and compared, as shown in Figure 14. It can be seen that the arrival time of the guided wave in the laboratory short rail test was basically consistent with the simulation results. Furthermore, based on the results from Figure 14, we can conclude that the propagation time of guided waves in a one-meter-long steel rail is approximately 0.323 s. Considering the actual distance between the transmitting and receiving transducers as 0.95 m, the velocity of guided wave propagation can be calculated as 2.94 m/s. In addition, the frequency was measured to be 35 kHz, which closely aligns with the velocity of the A0 wave indicated on the dispersion curve. This data strongly indicates that the observed guided wave corresponds to the A0 wave. Further comparison of the experimental and simulated signals revealed a high degree of similarity in their time-domain waveform and characteristics, demonstrating their consistency. The only difference was the width of the wave packet, which was due to the fact that the excitation current signal in the simulation was generated by a transient drive pulse, whereas in the actual excitation process, the oscillation of the current would cause an increase in the width of the small wave packet. In summary, the correctness of the finite element model for steel rail detection has been verified.

**Figure 15:**
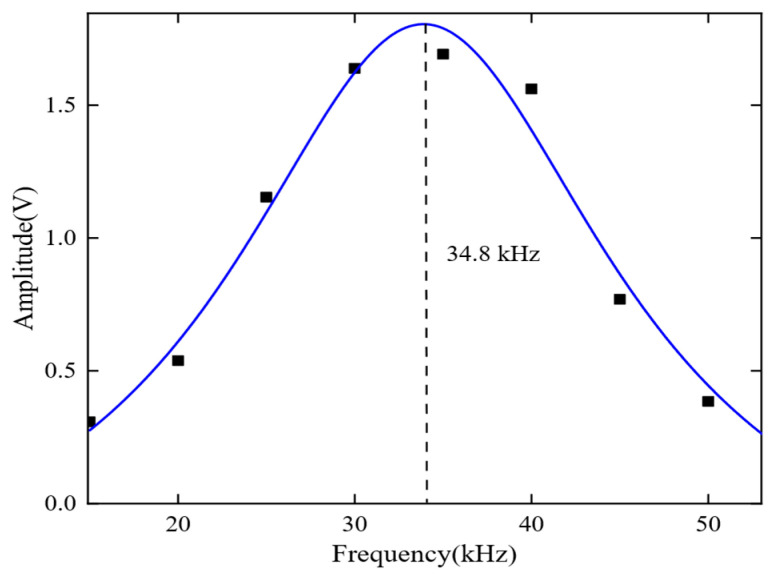
Frequency response characteristics of DMPS-EMAT.

During the experiment, the center distance between the transmitter and receiver was set to 1000 mm. The excitation frequency gradually increased from 15 kHz to 55 kHz in intervals of 5 kHz, and the signal amplitude was recorded at each frequency. The frequency response characteristic curve was plotted to display the experimental results, as shown in Figure 15. The red circles represent the signal amplitude at each frequency, while the blue curve is a normalized first-order Gaussian fit curve.

The results indicate that the DMPS-EMAT has the best frequency response characteristics, with an actual center frequency of 34.8 kHz and a relative error of 200 Hz compared to the theoretical center frequency of 35 kHz. This suggests that the designed EMAT exhibits excellent performance.

**Figure 16:**
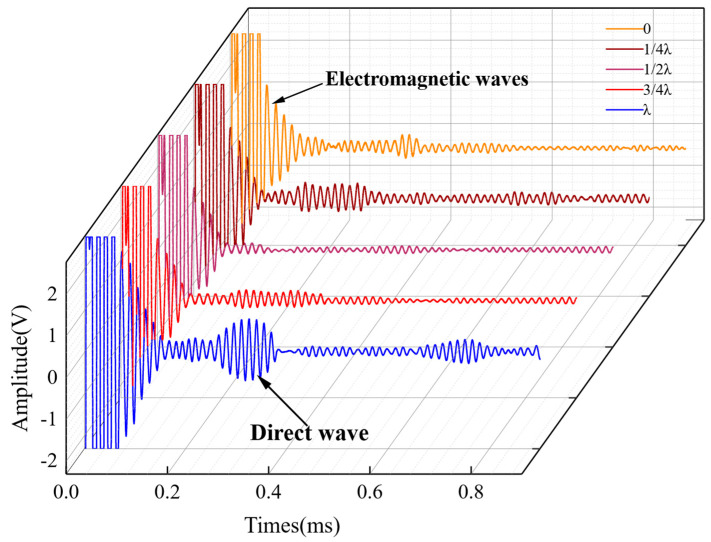
Waveforms of A0 waves generated by magnet distances at different wavelengths in the experiment.

To determine the appropriate distance between the double magnets for exciting a single-mode A0 Lamb wave, several experiments were conducted. In the experiment, a sensor was placed on a one-meter-long steel rail, and the distance between the magnets was varied. The results of the experiment are shown in Figure 16.

**Figure 17:**
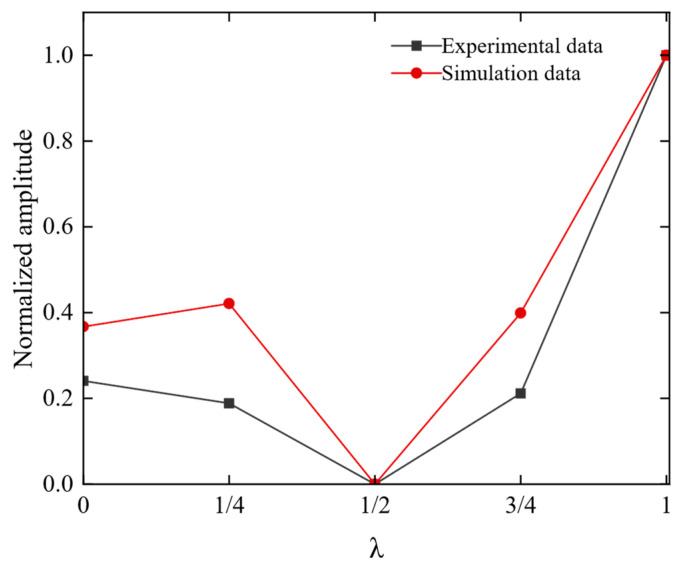
Effect of magnet distance of the different wavelengths on the amplitude of A0 waves in simulation and experiment.

To intuitively illustrate the variation of Lamb wave amplitude with respect to the distance between magnets, we plotted the amplitude changes of the A0 mode for different distances between magnets, as shown in Figure 17. It can be observed that the results from simulations and experiments are consistent: the peak-to-peak value of the guided wave reaches its maximum when the distance between magnets is an integer multiple of the wavelength. Conversely, when the distance between magnets is an odd multiple of half wavelength, the peak-to-peak value of the guided wave decreases due to destructive interference of waves. Therefore, to achieve the highest guided wave amplitude, the distance between magnets should be an integer multiple of the wavelength to achieve constructive interference. Meanwhile, choosing distances that are odd multiples of half-wavelength should be avoided to prevent destructive interference from reducing the guided wave amplitude.

**Figure 18:**
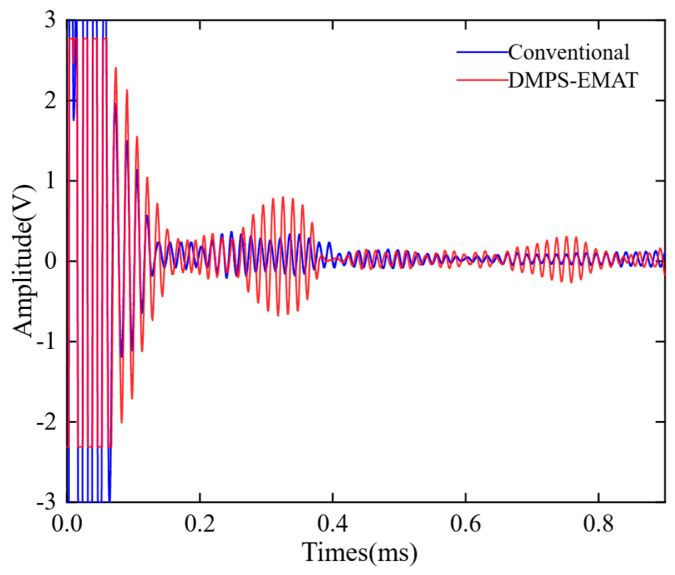
Comparison of improved and traditional electromagnetic ultrasonic receiving signals.

To verify the performance of the DMPS-EMAT, the A0 Lamb wave signals received by the traditional EMAT and the DMPS-EMAT were compared on a 1 m rail. As shown in Figure 18, the A0 Lamb wave signal obtained by DMPS-EMAT had no distortion in the time domain, and its amplitude was 2.35 times that of the conventional one. This result was attributed to the non-uniformly split wire coils and the waveform focusing structure adopted by the DMPS-EMAT. The experimental results were consistent with the simulation results shown in Figure 10, which confirmed that the DMPS-EMAT design effectively enhanced the A0 wave. Moreover, the larger signal amplitude of the A0 mode Lamb wave signal facilitated its identification from noise, indicating a significant improvement in the energy conversion efficiency of the new design, both theoretically and experimentally.

**Figure 19:**
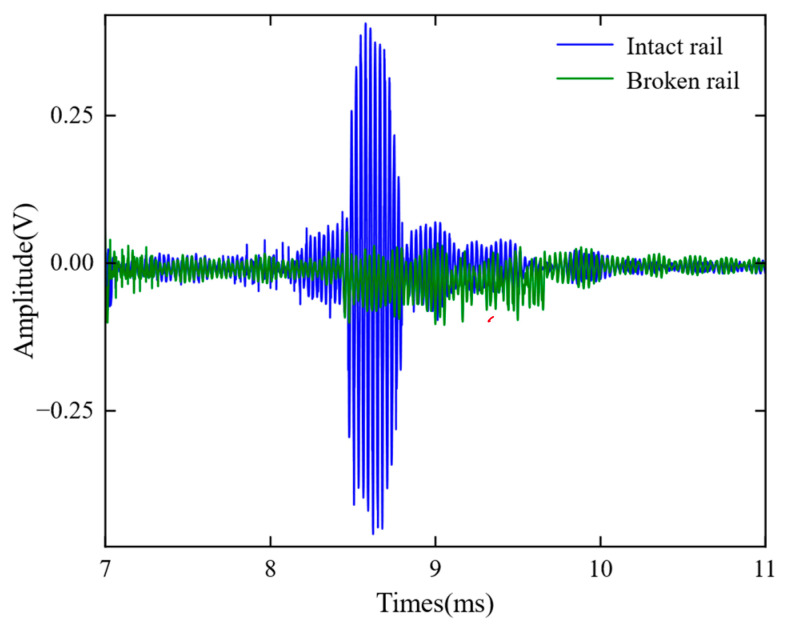
Waveforms captured before and after a rail fracture on a 25 m long rail using DMPS-EMAT.

In order to verify the feasibility of using DMPS-EMAT for remote detection of steel rails, a series of experimental studies were conducted. For these experiments, a 25 m section of rail was chosen for the transmit-receive testing. It was observed that the guided waves were able to propagate through the steel rail for nearly 25 m and still generate distinguishable pulse sequence signals. The received guided wave signals were amplified through the amplification circuit, reaching a peak-to-peak value of 0.84 V. These experimental results strongly demonstrate the feasibility of using DMPS-EMAT for remote detection of steel rails. To simulate a complete fracture of the rail, two rail sections were placed parallel at the joint. The distance between the transmitting and receiving ends was set to 25 m. Figure 19 illustrates the signals acquired before and after the rail fracture. When the rail is completely fractured, the received signal can be considered to have no input signal. In this scenario, the received signal can be measured and used as an estimation of the noise. Based on the experimental results, the signal-to-noise ratio at a distance of 25 m was determined to be approximately 11.29.

## 5. Conclusions

To excite a single-mode A0 Lamb wave in rails and improve the transduction efficiency of EMAT, this work proposes the DMPS-EMAT. This transducer enhances the A0 wave through constructive interference of guided waves, suppressing unwanted guided wave modes and increasing the magnetic flux density on the coil to generate a stronger Lorentz force in the transduced region. The finite element models were established to compare the performance of the DMPS-EMAT and the traditional EMAT under identical conditions regarding Lamb wave propagation and displacement detection. In our experimental research, we conducted a send-and-receive detection experiment on a 25 m rail and obtained a relatively clear pulse train signal after the wave propagated for almost 25 m. Additionally, the signal-to-noise ratio of the received signal at a distance of 25 m is approximately 11.29.

The simulation and experimental results indicate that the conversion efficiency of the DMPS-EMAT is 1.35 times higher than that of the traditional EMAT, demonstrating excellent performance. However, factors such as surface roughness, distance from the ground, and eddy currents may impact the robustness of the DMPS-EMAT and require further optimization and exploration in future research.

Traditional transducers face challenges such as low transduction efficiency and complex modal behavior. Additionally, they encounter limitations in terms of their large size and inconvenient installation when performing long-distance monitoring at low frequencies. In contrast, our designed transducer addresses these issues by improving the conversion efficiency and exciting a single-mode A0 guided wave. Furthermore, we have taken measures to minimize the volume of the low-frequency transducer. Additionally, the designed fixture allows for secure attachment of the transducer to the railway track, enabling long-distance, real-time detection in the rail.

## Figures and Tables

**Table 1:** Geometric parameters of EMAT.

	Parameter	Value
Coil	Width	0.1 mm
	Resistivity	1.7 × 10^−8^ mm
	List-off distance	0.1 mm
Magnet	Magnetic flux density	1.2 T
	Young’s modulus	209 GPa
	Possion´s ratio	0.29
Steel rail	Electrical conductivity	3.774 × 10^7^ (S/m)
	Density	60 kg/m^3^

## Data Availability

Data is available from the authors upon reasonable request.
